# The Relationship of Dioxin Levels in Serum of 9-Year-Old Vietnamese Children and Their Mothers’ Breast Milk

**DOI:** 10.3390/toxics10040155

**Published:** 2022-03-25

**Authors:** Ho Dung Manh, Teruhiko Kido, Takumi Takasuga, Michiko Yamashita, Le Minh Giang, Hideaki Nakagawa

**Affiliations:** 1 Faculty of Pharmacy, Lac Hong University, No. 10 Huynh Van Nghe, Buu Long, Bien Hoa 02513, Dong Nai, Vietnam; manhhodung@gmail.com; 2 Division of Health Sciences, Graduate School of Medical Science, Kanazawa University, 5-11-80 Kodatsuno, Kanazawa 920-0942, Japan; 3 Shimadzu Techno-Research Inc., 1 Nishinokyo Shimoaicho Nakagyo-ku, Kyoto 604-8436, Japan; t_takasuga00@shimadzu-techno.co.jp (T.T.); m_yamashita01@shimadzu-techno.co.jp (M.Y.); 4 10-80 Division, Hanoi Medical University, No. 1 Ton That Tung, Dong Da, Hanoi 116500, Vietnam; leminhgiang@hmu.edu.vn; 5 Department of Hygiene, Kanazawa Medical University, 1-1 Daigaku, Uchinada, Kahoku 920-0265, Japan; hnakagaw@kanazawa-med.ac.jp

**Keywords:** dioxin, Agent Orange, Vietnam, children

## Abstract

In this study, we measured the concentrations of polychlorinated dibenzo-p-dioxins (PCDDs) and polychlorinated dibenzofurans (PCDFs) in the blood of 9-year-old children living in a dioxin hotspot area and a nonexposed area in Vietnam. Forty-five blood samples were collected in the hotspot area while twelve pooled blood samples were collected in the nonexposed area. We found that the dioxin level of children in the hotspot was significantly higher than that of children in the nonexposed area. The total TEQ of PCDD/Fs in the hotspot and the nonexposed was 10.7 and 3.3 pg TEQ/g fat, respectively. However, TCDD, the maker of Agent Orange, was not detected in the blood of children in the hotspot area. In the hotspot area, four congeners 1,2,3,4,6,7,8-HpCDD, 1,2,3,4,7,8-HxCDF, 1,2,3,6,7,8-HxCDF, and 1,2,3,4,6,7,8-HpCDF in mothers’ breast milk showed a significantly positive correlation with those in children’s serum although the correlations of 1,2,3,7,8-PeCDD and 2,3,4,7,8-PeCDF were not significant. In addition, the duration of breastfeeding also correlates with dioxins in children. These results suggested that children in the hotspot area were exposed to dioxin through mothers’ milk and other foods or environmental factors. The present study is the first study that shows dioxin levels in Vietnamese children.

## 1. Introduction

During Operation Ranch Hand (1961−1971), the U.S. military sprayed millions of liters of herbicide Agent Orange over Southern Vietnam [1]. Unfortunately, the herbicide was contaminated with 2,3,7,8-tetrachlorodibenzo-p-dioxin (TCDD), which is one of the most toxic chemicals of the dioxins and is classified as a human carcinogen by the U.S. Environmental Protection Agency (EPA). More than 40 years have passed, and the dioxin levels are not very high in herbicide-sprayed areas due to degradation over a long time [2]. However, high dioxin levels have been still found in the soil inside former U.S. air bases as a result of herbicide spills, washing out herbicide tanks, and high-volume ground applications. These former air bases are now known as dioxin hotspots in Vietnam [3,4,5]. Recent studies on these hotspots have found that dioxin levels were very high in environmental and food samples [6]. In addition, the dioxin levels in breast milk and blood of Vietnamese peoples living around dioxin hotspot areas are also much higher than people living in nonexposed areas [2,7,8,9,10,11,12,13,14,15].

There is a growing concern about continuous dioxin exposure for Vietnamese people who live around dioxin hotspots. Recent studies have focused on the health effects of dioxin on children’s development living around dioxin hotspots. These studies have shown that dioxin exposure has effects on endocrine disruption, physical development, and neurodevelopment in children [12,16,17,18,19,20,21]. However, these studies usually assess dioxin exposure in children by measuring dioxin levels in their mother’s breast milk after the children are born. In this study, we will measure dioxin exposure in children by analyzing dioxin in their blood samples.

Several studies have shown that postnatal exposure to dioxins induces adverse effects on children. Studies in Chapaevsk, Russia have shown the association of peripubertal serum concentrations of organochlorines with growth, pubertal onset, and sexual maturity [22]. Studies in Seveso, Italy have shown that dioxin exposure in infancy/prepuberty lead to permanent effect on semen quality in human males as a result of the disruptive action of low concentrations of TCDD on the endocrine system [23].

To our knowledge, there is no study that has reported dioxin levels in children living in the hotspot areas in Vietnam. This may be due to difficulty in obtaining samples and the analytical technique. In this study, we aimed to measure and compare dioxin levels in blood of the 9-year-old children living in a dioxin hotspot area and a nonexposed area as well as assess the relationship between dioxin levels in children and their mother’s breast milk.

## 2. Materials and Methods

### 2.1. Study Areas

**Figure 1:**
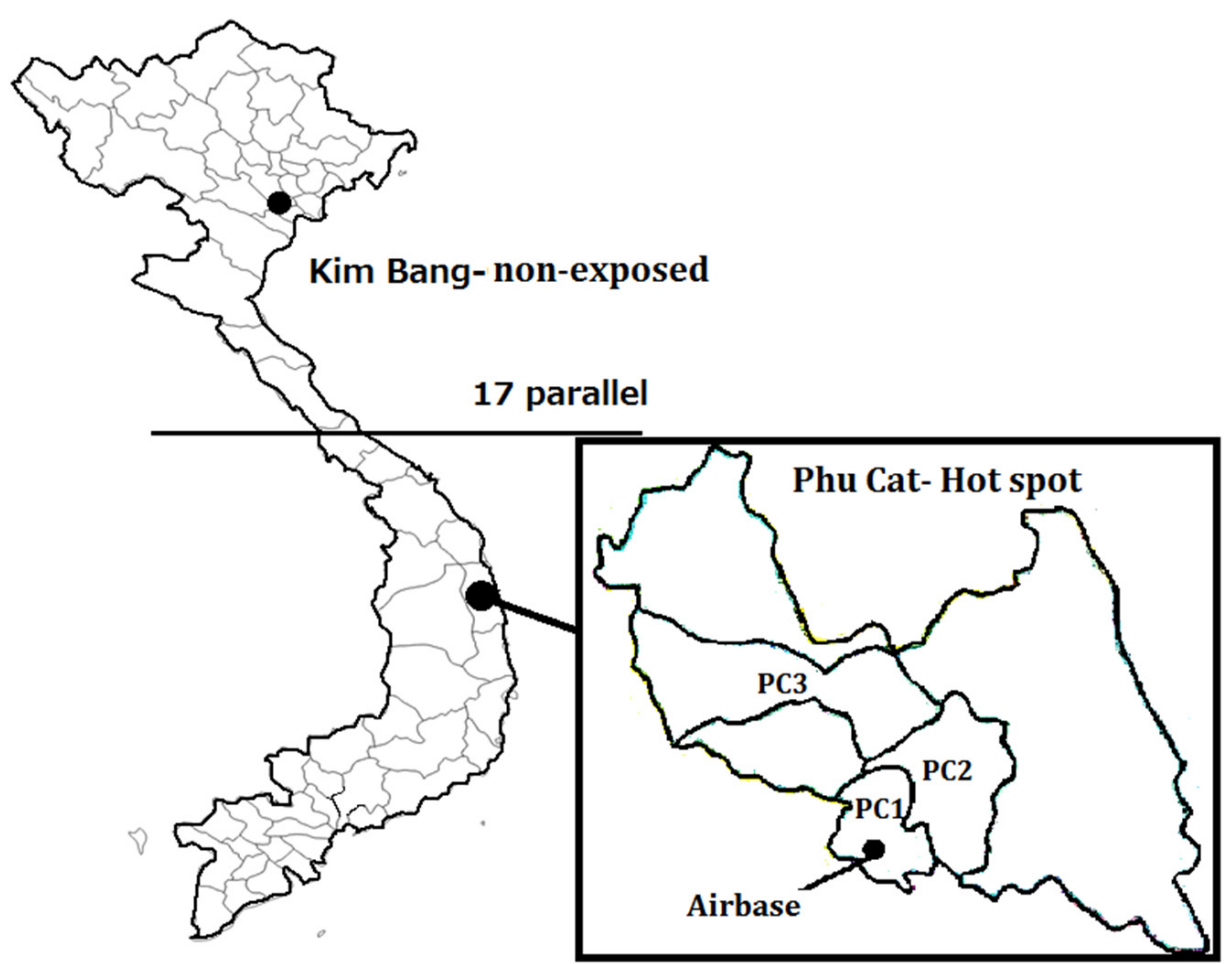
Study areas in Vietnam. The 17 parallel is the dividing line between North Vietnam and South Vietnam, as established by the 1954 Geneva Conference. PC1, PC2, and PC3 represent three subareas in the Phu Cat hotspot.

The study areas are shown in Figure 1. Phu Cat Air Base is one of the three most dioxin-contaminated former U.S. air bases [5]. This air base is located in Phu Cat district, a rural area in Binh Dinh province. The TCDD level in the soil inside the air base has been found to be at a very high level of 236,000 pg/g taken at the herbicide storage area inside the air base [24], which suggests significant involvement of Agent Orange herbicide in soil/sediment samples because TCDD was the characteristic dioxin congener in Agent Orange. In the Phu Cat district, three subareas (PC1, PC2 and PC3) were selected according to their distance from the air base. The air base is inside PC1 (the Cat Tan and Ngo May communes, total area 40 km^2^), near PC2 (Cat Tuong and Cat Trinh communes, total area 80 km^2^), and a little farther from PC3 (Cat Hanh and Cat Lam communes, total area about 110 km^2^).

The nonexposed area is Kim Bang district, which is located in Ha Nam province, in northern Vietnam. This area is also a rural area without an industrial zone nearby.

### 2.2. Study Participants

The initial study started in 2008. We collected breast milk from 60 mothers in the hotspot and 63 mothers from the nonexposed area when these children were 4–16 weeks [12]. In 2017, 45 blood samples of 9-year-old children were collected in the hotspot area and 35 blood samples of 9-year-old children were collected in the nonexposed area. The causes of loss in this follow-up study in both areas were due to traveling, visiting relatives, illness, or parent refusal. In the nonexposed area, due to the low level of dioxin, we combined each group of several samples to make a total of 12 pooled samples. In mother–child pair samples, we missed one breast milk sample from the hotspot and two breast milk samples from the nonexposed area. In this study, all mothers reported that their children were breastfed for 5 months on average (range from 1–9 months).

The purpose of the present study was thoroughly explained to them and written informed consent was obtained from each participant through their local people’s committee. This study was approved by the Medical Ethics Committee of Kanazawa University (No. 455, 12 July 2013).

### 2.3. Dioxin Analysis

About 5 mL of blood were collected from each participant by medical staff at the community health centers. We then separated serum from samples and kept it frozen before sending it to Shimadzu Techno-Research Inc., in Kyoto, Japan for analysis. The analysis of 7 polychlorinated dibenzodioxins (PCDDs) and 10 polychlorinated dibenzofurans (PCDFs) in serum were described in the previous report [9]. After extraction and purification, the dioxins were quantified using high-resolution gas chromatography–high-resolution mass spectrometry (Hewlett-Packard 6890 Series and Micromass Autospec, Ultima). All PCDD/Fs congeners were calculated on a lipid basis which was determined from the crude extract by gravimetric method [25], then converted to toxic equivalents (TEQs) using the international toxicity equivalency factors (TEFs) 2005 recommended by the World Health Organization (WHO) [26]. The detection limits of congeners varied depending on the samples. Generally, a serum sample of 2.0 g wet weight with 0.5% lipid content (*w*/*w*) has detection limits, as follows: Te-PeCDD/Fs: 0.02 pg/g wet, 3 pg/g lipid; Hx-HpCDD/Fs: 0.04 pg/g wet, 6 pg/g lipid; OCDD/Fs: 0.1 pg/g wet, 10 pg/g lipid. Serum concentrations of dioxin and furan congeners below the limit of detection (LOD) were assigned a value equal to half the LOD.

Dioxin analysis in mothers’ milk was described in our previous report [10]. Briefly, lipids were extracted from 10 g breast milk by liquid extraction and spiked with 40–80 pg of seventeen ^13^C_12_-labeled PCDD/F congeners, as an internal standard. PCDD/Fs were purified on a multi-layer silica gel column and separated by an active carbon-dispersed silica gel column. The final sample extract was evaporated to dryness under a nitrogen stream then redissolved by addition of 20 μL of nonane containing 40 pg of ^13^C_12_-1,2,3,4-TCDD and ^13^C_12_-1,2,7,8-TCDF as external standards. PCDD/Fs were quantified using a gas chromatograph (HP-6980, Hewlett-Packard, Palo Alto, CA, USA) equipped with a high-resolution mass spectrometer (HRMS: JEOL MS station—JMS700). Analyses were performed in the selected ion-monitoring mode, and the resolution was maintained above 10,000. Seventeen PCDD/F congeners were calculated on a lipid basis and then converted to TEQ using the World Health Organization toxicity equivalency factors 2005. The detection limits were determined at a signal-to-noise ratio of 3. Samples with undetectable congener concentrations were assigned a value equal to half the LOD.

### 2.4. Data Analysis

We used R Statistical Environment (R Development Core Team, 2013) and Microsoft Excel 2010 (Microsoft Corp., Redmond, WA, USA) to conduct statistical analyses. Data are shown as geometric mean. The mean difference of each indicator between two areas or two genders was calculated by applying either Student’s *t*-test or the Mann–Whitney U-test, depending on their distribution, as judged by a Shapiro–Wilk test. For comparison among three subareas in the hotspot, ANOVA test was used. Spearman’s rank correlations were calculated between the concentrations of PCDD/F congeners in mothers’ breast milk and the children’s serum. The significance level was set to *p* < 0.05. Multiple linear regression was used to find the relationship of dioxin in the blood of children and dioxin in mother’s breast milk, duration of breastfeeding, and children’s gender. This information was obtained by interviewing the mothers during sample collection.

## 3. Results and Discussion

### 3.1. Serum Concentration of PCDD/Fs in Children

Table 1 shows dioxin levels in children in both areas. Only dioxin and furan congeners detected are shown. Four PCDD congeners, including 1,2,3,7,8-PeCDD, 1,2,3,6,7,8-HxCDD, 1,2,3,4,6,7,8-HpCDD, and OCDD are detected in both areas. The levels of these four PCDD congeners were also significantly higher in the hotspot than the nonexposed area. The other three PCDD congeners, including 2,3,7,8-TeCDD, 1,2,3,4,7,8-HxCDD, and 1,2,3,7,8,9-HxCDD, were below LOD in all samples.

Four PCDF congeners, including 2,3,4,7,8-PeCDF, 1,2,3,4,7,8-HxCDF, 1,2,3,6,7,8-HxCDF, and 1,2,3,4,6,7,8-HpCDF were detected in the hotspot area while only three congeners, 2,3,4,7,8-PeCDF, 1,2,3,4,7,8-HxCDF, and 1,2,3,6,7,8-HxCDF, were detected in lower percentage of samples in the nonexposed area. In particular, 1,2,3,4,6,7,8-HpCDF levels were detected in all samples in the hotspot while not detected in any sample in the nonexposed area. The levels of these PCDF congeners were also significant higher in the hotspot than the nonexposed area. Other PCDF congeners were not detected in both areas.

Table 2 shows dioxin levels in the hotspot area divided by gender and subareas. The proportion of males/females in the hotspot is 26/18, and in nonexposed is 13/22. This number is not similar in the two areas, but we did not find any difference in dioxin levels between males and females in the hotspot. In addition, there is no difference in dioxin levels among the children in the three subareas.

TCDD is the characteristic dioxin congener in Agent Orange. However, no sample has a TCDD level higher than its LOD. Previous studies have found TCDD in mothers’ breast milk and blood of old men in the hotspot area around Phu Cat Air Base [9,10]. Furthermore, these studies also found that dioxin levels are significantly correlated with residential years. In this study, the children are only 9 years old. Besides dioxin exposure through breast milk, their exposure time to the external environment is short. In addition, when comparing dioxin levels in mothers’ breast milk in three hotspots in Vietnam, we found that TCDD in breast milk in Phu Cat is low (range of 1–3 pg/g lipid) as compared with TCDD in breast milk samples collected in Bien Hoa (range of 0–27 pg/g lipid) or Da Nang (range of 1–10 pg/g lipid) hotspot [10]. One of the reasons is that people in Phu Cat live far from the air base, while in Bien Hoa city, people live near the air base. Therefore, it is necessary that we also biomonitor dioxin levels in children living in other hotspot areas such as Bien Hoa or Da Nang air base in future studies.

The children’s serum concentration of total TEQ PCDD/Fs was 10.7 pg/g lipid in the hotspot, while it was 3.3 pg/g lipid in the nonexposed area. In Chapaevsk of Russia, an area with an extensive chemical manufacturing industry, the Khimprom chemical plant has produced chlorine-containing industrial and agricultural chemicals. The children of 8–9 years of age in this area have a median of TEQ PCDD/Fs level as high as 12.4 pg/g lipid in the blood samples collected between 2003–2005 [27]. In Kita borough of Tokyo, Japan where dioxin in the soil at the residential area is detected at a high level, the dioxin in the blood of 33 children (20 males and 13 females) whose age ranged from 7 to 15 years collected in 2006 is also high, with a mean of 5.5 pg/g lipid [28]. In German study, 10-year-old children living in industrial regions show a decreasing dioxin level. In 1993–1994, the average TEQ of PCDD/Fs was 10.2 pg/g lipid, and this level has decreased over time. In the most recent period of 2002–2003, the average of TEQ PCDD/Fs was reduced to 5.5 pg/g lipid [29]. There are a few studies worldwide that report dioxin levels in the blood of children, and therefore our study has added some valuable data on children’s dioxin levels in the world.

### 3.2. Correlation of Dioxin in Children’s Blood and Mothers’ Breast Milk

Table 3 shows the correlation of dioxin in children and their mothers in the hotspot and the nonexposed area. In the hotspot area, four congeners, 1,2,3,4,6,7,8-HpCDD, 1,2,3,4,7,8-HxCDF, 1,2,3,6,7,8-HxCDF, and 1,2,3,4,6,7,8-HpCDF showed significant correlations between mothers’ breast milk and children’s serum. However, the correlations of 1,2,3,7,8-PeCDD, 2,3,4,7,8-PeCDF, and TEQ of PCDDs, PCDFs, or PCDD/Fs in mothers’ milk and children’s blood were not significant. This might be explained based on congeners’ TEF (toxic equivalent factor), which is used to calculate TEQ. TEF of 1,2,3,7,8-PeCDD is 1 and is relatively high compared with those of HxCDD 0.1, HpCDD 0.01, and OCDD 0.0003 [26]. PCDFs also show a similar trend, with the highest TEF of 2,3,4,7,8-PeCDF being 0.3. This means that the PeCDD or PeCDF highly affect TEQ of PCDD, PCDF, and PCDD/Fs. Therefore, when the correlation of PeCDD and PeCDF was a very low value of −0.073 and 0.075, the correlations of PCDD, PCDF, and PCDD/Fs were also low and not significant. In addition, OCDD did not show a significant correlation in the hotspot. A previous study showed that OCDD levels are very high in food samples compared to other dioxin congeners [30]. The children are exposed to OCDD from both food intake and breast milk. Therefore, OCDD levels in children’s blood did not correlate well with OCDD levels in their mother’s milk in the hotspot.

In the nonexposed area, we have used pooled samples with an equal blood volume of several children. Therefore, we calculated the mean of dioxin levels in their mothers’ breast milk samples. In the nonexposed area, most dioxin/furan congeners did not show a significant correlation between dioxin in mothers’ milk and children’s blood, but TEQ of PCDDs, PCDFs, and PCDD/Fs showed significant correlations. As we can see in Table 3, the correlation coefficients of most congeners were relatively high. For example, the correlation coefficients of 1,2,3,7,8-PeCDD and 2,3,4,7,8-PeCDF were 0.461 and 0.467, but their *p*-values were not significant. One reason might be due to the small number of samples (n = 10). However, because most individual congeners showed a similar trend of the high positive value of correlation coefficients, their TEQ values showed significant correlations even with a small number of samples.

Table 4 shows the relationship of dioxin level in children’s serum and their gender, duration of full breastfeeding, and dioxin level in breast milk in the hotspot area assessed by using linear multiple regression. Gender shows no correlation with dioxin levels. Four dioxin congeners, 1,2,3,4,6,7,8-HpCDD, 1,2,3,4,7,8-HxCDF, 1,2,3,6,7,8-HxCDF, and 1,2,3,4,6,7,8-HpCDF, in breast milk have significant correlation with dioxin levels in children’s serum. In addition, 2,3,4,7,8-PeCDF, 1,2,3,6,7,8-HxCDF, and PCDFs TEQ show correlation between dioxin in children’s serum and duration of breastfeeding. In the nonexposed area, we could not assess such association due to pooled samples, not individual samples.

The correlation of dioxin in children and their mothers suggested that one source of dioxin exposure in children is their mother’s breast milk. In Vietnam, most children drink breast milk from when they are born. In this study, all mothers reported that their children were breastfed for 5 months on average (range from 1–9 months). The results are similar to the Russia study where 8- to 9-year-old boys who were breastfed for 26 weeks had a 28% increase in serum concentration compared with boys who were not breastfed [27]. In Kita borough, a dioxin-contaminated area in Tokyo, Japan, dioxin levels in 3- to 15-year-old children who were breastfed are about threefold higher than children that used formula milk. This study also found an association between the duration of breastfeeding and dioxin level in children [28]. In Germany, 10-year-old children living in industrial areas have 30% higher TEQ PCDD/Fs in the breastfeeding group than the non-breastfeeding group [29]. These studies only analyzed dioxin levels in children. However, our study measured dioxins in both children and mothers’ breast milk and confirmed the dioxin exposure through mother’s breastfeeding. Besides dioxin exposure through breast milk in their early stages, children are also exposed to dioxin through food intake. Food with high dioxin levels, such as free-range chicken meat and eggs, ducks, freshwater fish, snails, and beef, were reported in another hotspot area [31].

### 3.3. Comparison of Dioxin Congener Patterns in Children, Mothers, and Men in the Hotspot Area

**Figure 2:**
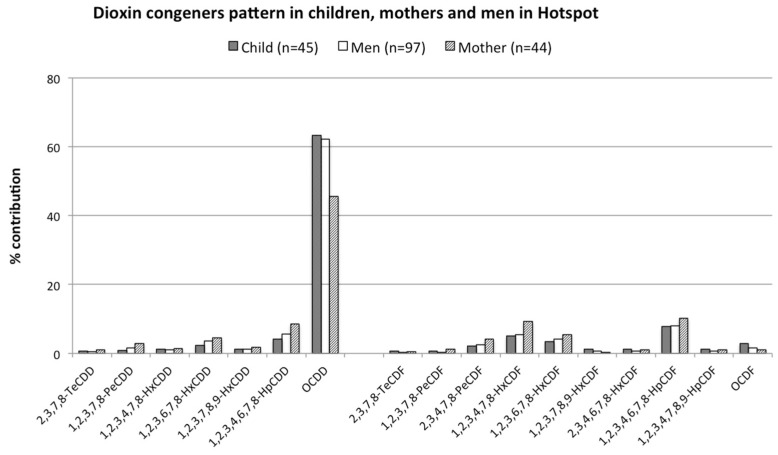
Congener patterns for PCDD/Fs in children, mothers, and men living in the hotspot area.

We also compare the dioxin congener patterns in the serum of children and men and mothers’ milk in the hotspot area. The congener pattern is expressed as a relative contribution to the sum of the measured concentrations. The serum samples of old men (mean age of 68 years old) who lived in this area were also collected in 2010–2011, and dioxin levels were described in the previous report [11]. Figure 2 shows a similar congener pattern between dioxin in children, mothers, and men. OCDD shows the highest contribution in men (62.1%), mothers (46.0%), and children (63.3%). Other PCDDs show the main contributions are 1,2,3,7,8-PeCDD, 1,2,3,6,7,8-HxCDD, 1,2,3,4,6,7,8-HpCDD, while four PCDFs show the major contributions are 2,3,4,7,8-PeCDF, 1,2,3,4,7,8-HxCDF, 1,2,3,6,7,8-HxCDF, and 1,2,3,4,6,7,8-HpCDF.

Recent dioxin decontamination projects were completed in Phu Cat Air Base where more than 7500 m^3^ of dioxin-contaminated soil were contained in landfills in 2012 [32]. Therefore, the young generation may have little exposure to TCDD from the air base. Children are usually not exposed to occupational pollution and therefore represent a better trend of external exposure than adults. In the previous study, we found that men who live in PC1 (closest to the air base) have the highest dioxin levels; however, in this study, we do not find any difference in children in the three subareas. One reason might be that men also have occupational exposure, since in the previous study we found that TCDD was highest in two men (16 and 25 pg/g lipid) who had worked inside the air base [11]. Future studies on monitoring trend of dioxin levels in children will be necessary to reconfirm the effects of these remediation actions.

## 4. Conclusions

To our best knowledge, this is the first report showing dioxin levels in Vietnamese children living around a hotspot area. We found that dioxin levels of children living in the hotspot were significantly higher than those in the nonexposed area. The dioxin levels in children were correlated with dioxin level in mothers’ breast milk and duration of breastfeeding. Future studies should focus on the health effects of dioxin on children and expand biomonitoring dioxin levels in children living in other hotspots in Vietnam.

## Figures and Tables

**Table 1:** Dioxin levels in serum of children in the hotspot and the nonexposed areas.

pg/g Lipid	Hotspot (*n* = 45, Individual Samples)				Nonexposed (*n* = 12, Pooled Samples)				
	% over LOD	GM	GSD	Median	% over LOD	GM	GSD	Median	*p*-Value
**PCDD congeners**									
1,2,3,7,8-PeCDD	44	2.3	1.7	2.0	33	1.0	1.4	1.0	<0.0001 ^a^
1,2,3,6,7,8-HxCDD	60	6.0	1.8	7.0	8	1.6	1.1	1.5	<0.0001 ^a^
1,2,3,4,6,7,8-HpCDD	91	10.7	1.8	10.0	58	2.5	1.6	3.0	<0.0001 ^a^
OCDD	100	161.6	1.6	160.0	100	64.3	1.3	63.0	<0.0001 ^a^
**PCDF congeners**									
2,3,4,7,8-PeCDF	98	5.6	1.5	6.0	92	2.5	1.6	3.0	<0.0001 ^a^
1,2,3,4,7,8-HxCDF	96	12.8	1.7	12.0	8	1.6	1.1	1.5	<0.0001 ^a^
1,2,3,6,7,8-HxCDF	87	8.9	1.8	9.0	8	1.6	1.1	1.5	<0.0001 ^a^
1,2,3,4,6,7,8-HpCDF	100	19.7	1.8	18.0	0	1.5	1.2	1.5	<0.0001 ^a^
**TEQ pg/g lipid**									
TEQ PCDDs		5.5	1.4	5.3		1.8	1.3	1.5	<0.0001 ^a^
TEQ PCDFs		5.1	1.4	4.7		1.6	1.3	1.6	<0.0001 ^a^
TEQ PCDD/Fs		10.7	1.3	10.0		3.3	1.2	3.1	<0.0001 ^a^

Note: n: number of subjects; GM: geometrical mean; GSD: geometrical standard deviation; LOD: limit of detection, ^a^ Mann–Whiney test.

**Table 2:** Geometric mean of dioxin levels in hotspot divided by gender and three subareas.

pg/g Lipid	Gender		*p* Value ^a^	Subareas			*p* Value ^b^
	Male (n = 26)	Female (n = 18)		PC1 (n = 18)	PC2 (n = 15)	PC3 (n = 11)	
**PCDD congeners**							
1,2,3,7,8-PeCDD	2.2	2.6	0.2	2.3	2.2	2.6	0.9
1,2,3,6,7,8-HxCDD	5.9	6.1	0.7	5.7	5.3	7.4	0.2
1,2,3,4,6,7,8-HpCDD	11.0	10.0	0.4	11.2	9.8	10.8	0.8
OCDD	163.5	153.8	0.7	158.6	165.0	153.5	1.0
**PCDF congeners**							
2,3,4,7,8-PeCDF	5.9	5.1	0.4	5.4	5.3	6.5	0.4
1,2,3,4,7,8-HxCDF	13.4	11.7	0.3	11.8	11.5	16.3	0.1
1,2,3,6,7,8-HxCDF	10.0	7.5	0.1	8.6	7.6	11.9	0.1
1,2,3,4,6,7,8-HpCDF	19.7	19.6	0.7	18.0	20.1	22.0	0.3
**TEQ pg/g lipid**							
TEQ PCDDs	5.3	5.7	0.5	5.2	5.3	6.1	0.5
TEQ PCDFs	5.3	4.7	0.1	4.7	4.8	6.1	0.1
TEQ PCDD/Fs	10.7	10.6	1.0	10.0	10.3	12.3	0.1

Note: n: number of subjects; ^a^ Mann–Whiney test; ^b^ ANOVA test.

**Table 3:** Spearman’s rank correlation between the concentrations of PCDD/F congeners in mothers’ breast milk and the children’s serum.

	Hotspot (n = 44)		Nonexposed (Pooled Samples, n = 10)	
	r	*p*	r	*p*
**PCDD congeners**				
1,2,3,7,8-PeCDD	−0.073	0.637	0.461	0.180
1,2,3,6,7,8-HxCDD	0.159	0.303	−0.115	0.753
1,2,3,4,6,7,8-HpCDD	0.402	**0.007**	0.370	0.293
OCDD	0.140	0.365	0.675	**0.032**
**PCDF congeners**				
2,3,4,7,8-PeCDF	0.075	0.628	0.467	0.173
1,2,3,4,7,8-HxCDF	0.410	**0.006**	0.306	0.390
1,2,3,6,7,8-HxCDF	0.333	**0.027**	0.309	0.386
1,2,3,4,6,7,8-HpCDF	0.440	**0.003**	0.090	0.804
PCDDs TEQ	−0.047	0.763	0.663	**0.037**
PCDFs TEQ	0.237	0.121	0.642	**0.045**
PCDD/Fs TEQ	−0.028	0.854	0.705	**0.023**

**Table 4:** Relationship of dioxin level in children’s serum and their gender, duration of full breast feeding, and dioxin level in breast milk in the hotspot assessed by using linear multiple regression.

	Dioxin in Breast Milk (β)	Gender (β)	Duration of Breast Feeding (β)	R2
**PCDD congeners**				
1,2,3,7,8-PeCDD	−0.034	0.174	0.189	0.063
1,2,3,6,7,8-HxCDD	0.197	0.178	0.298	0.106
1,2,3,4,6,7,8-HpCDD	0.321 *	0.054	0.209	0.143
OCDD	0.133	0.003	0.147	0.042
**PCDF congeners**				
2,3,4,7,8-PeCDF	0.035	0.102	0.378 *	0.170
1,2,3,4,7,8-HxCDF	0.460 **	0.115	0.261	0.226
1,2,3,6,7,8-HxCDF	0.372 *	−0.007	0.381 **	0.280
1,2,3,4,6,7,8-HpCDF	0.426 **	0.173	0.257	0.209
TEQ PCDDs	−0.106	0.104	0.214	0.072
TEQ PCDFs	0.221	−0.006	0.339 *	0.158
TEQ PCDD/Fs	−0.083	−0.021	0.278	0.086

Note: n = 44; β: standardized regression coefficient, * *p* < 0.05, ** *p* < 0.01.

## Data Availability

The data presented in this study are available on request to the corresponding author.
